# Coffee Drinking

**Published:** 1891-01

**Authors:** 


### COFFEE DRINKING.

Caffeine has not, as is generally believed, the marvelous property of replacing food ; it only replaces the general tonic excitation which the ingestion of food produces. It is admitted that it is the direct and instantaneous action of the aliments which stimulate the stomach and nervous system, and that their alimentary value is at first nothing; one might substitute one stimulant for another. Caffeine, far from sparing the reserves, will place a fasting man in a position to undertake his work only by attacking these reserves, the destruction of which it hastens by the excitation of the nervous system, and by its medium that of the muscles ; the organism will then soon use up its nutritive supply, and the caffeine will not prevent it. It is, nevertheless, of incontestable, but temporary, utility for the physical forces.

